# Successful Treatment of Risperidone-Induced Hypothermia With Aripiprazole: A Brief Case Report and a Literature Review

**DOI:** 10.7759/cureus.66464

**Published:** 2024-08-08

**Authors:** Ahmad Obeidat, Feras Al-Moussally, Waseem Abdallah

**Affiliations:** 1 Department of Internal Medicine, MedStar Washington Hospital Center, Washington, DC, USA; 2 Internal Medicine, University of Central Florida College of Medicine, Orlando, USA; 3 Department of Psychiatry, MedStar Washington Hospital Center, Washington, DC, USA

**Keywords:** schizophrenia, aripiprazole, risperidone, antipsychotics, hypothermia

## Abstract

Hypothermia, a potentially fatal condition, can result from various internal causes, including certain medications. Antipsychotics, in particular, are associated with hypothermia, typically emerging 7-10 days after dosage adjustments. Here, we report the case of a 68-year-old male with a history of cerebral palsy, bipolar disorder, and paranoid schizophrenia who was admitted due to poor oral intake and was found to have persistent hypothermia despite active external rewarming. After substituting risperidone with aripiprazole, his temperature normalized, and he experienced no further hypothermic episodes.

Antipsychotic-induced hypothermia should be considered in patients on these medications who present with non-specific symptoms. Regular monitoring of temperature and vital signs is crucial for early detection and management.

## Introduction

Antipsychotic medications are commonly prescribed to address various psychiatric conditions, playing a crucial role in symptom management and enhancing the overall well-being of patients. However, emerging evidence suggests a link between the use of antipsychotics and the occurrence of hypothermia - a phenomenon not frequently documented in medical literature. The exact pathophysiology of such a phenomenon continues to be unclear. However, it is hypothesized that such a side effect occurs due to a combination of multiple interactions and might be a little different depending on the exact antipsychotic ​[1,2]. In the case of atypical antipsychotics, it is speculated that hypothermia might be the result of antagonism of the 5-HT2 receptor which alters thermoregulation, in addition to alpha-adrenergic receptor antagonism which results in inhibition of peripheral response to cooling ​[2,3]. 

The diagnosis and management of hypothermia can be done on the basis of the Swiss staging system ​[4], or according to the traditional staging (mild, moderate, severe, and profound hypothermia) ​[5]. After addressing the hypothermia according to the guidelines, managing antipsychotic-related hypothermia might necessitate stopping the medication ​[6]. 

The association between antipsychotics and hypothermia has been reported in the literature to be highest within 7 to 10 days of initiating or adjusting medication doses [6]. This case report presents a compelling scenario in which hypothermia manifested after a stable year-long course of antipsychotic therapy without recent dose adjustments. This underscores the importance of vigilant monitoring for potential side effects and adjusting dosage as necessary, emphasizing the significance of understanding and addressing this potentially life-threatening complication. 

## Case presentation

A 68-year-old male with a medical history of cerebral palsy, bipolar disorder, schizophrenia, congenital right hemiparesis, epilepsy, and prostate cancer status post-radiation was admitted for poor oral intake for several days. 

Upon presentation to the emergency department, the patient had hypoglycemia with a glucose level of 53 mg/dL, hypothermia with a core body temperature of 34.6°C, and sinus bradycardia ranging from 40 to 50 beats per minute, accompanied by a normal blood pressure of 128/68 mmHg. Passive external rewarming failed, so the patient was admitted to the critical care unit for active external rewarming (AER). The patient's laboratory results on admission are given in Table 1. 

**Table 1:** The patient’s laboratory results on presentation

Lab	Patient's value	Reference range
White Blood Cell (WBC)	3.33 k/mm3	4.5-11.0 k/mm3
Hemoglobin	12.4 g/dL	13.5-17.5 g/dL
Platelets	135 k/mm3	150-400 k/mm3
Morning Cortisol	3 μg/dL	6-23 μg/dL
Thyroid-stimulating Hormone (TSH)	1.7 μIU/m	0.4-4.0 μIU/mL
Aspartate Transaminase (AST)	73 units/L	10-40 units/L
Alanine Transaminase (ALT)	128 units/L	10-49 units/L

A subsequent adrenocorticotropic hormone (ACTH) stimulation test was done and revealed an appropriate response with cortisol levels of 32 mcg/dL, 46 mcg/dL, and 39 mcg/dL at 30, 60, and 90 minutes, respectively (normal response: cortisol levels should increase by at least 18-20 mcg/dL from baseline and reach a peak level of at least 18-20 mcg/dL). 

The patient continued his home medications, including risperidone (3 mg every morning and 4 mg every evening), which had remained stable for the past year without recent adjustments. Additionally, he was on a stable dose of lacosamide and levetiracetam with no reported seizure activity, and empiric antibiotics therapy was initiated due to a concern for sepsis. 

**Figure 1:**
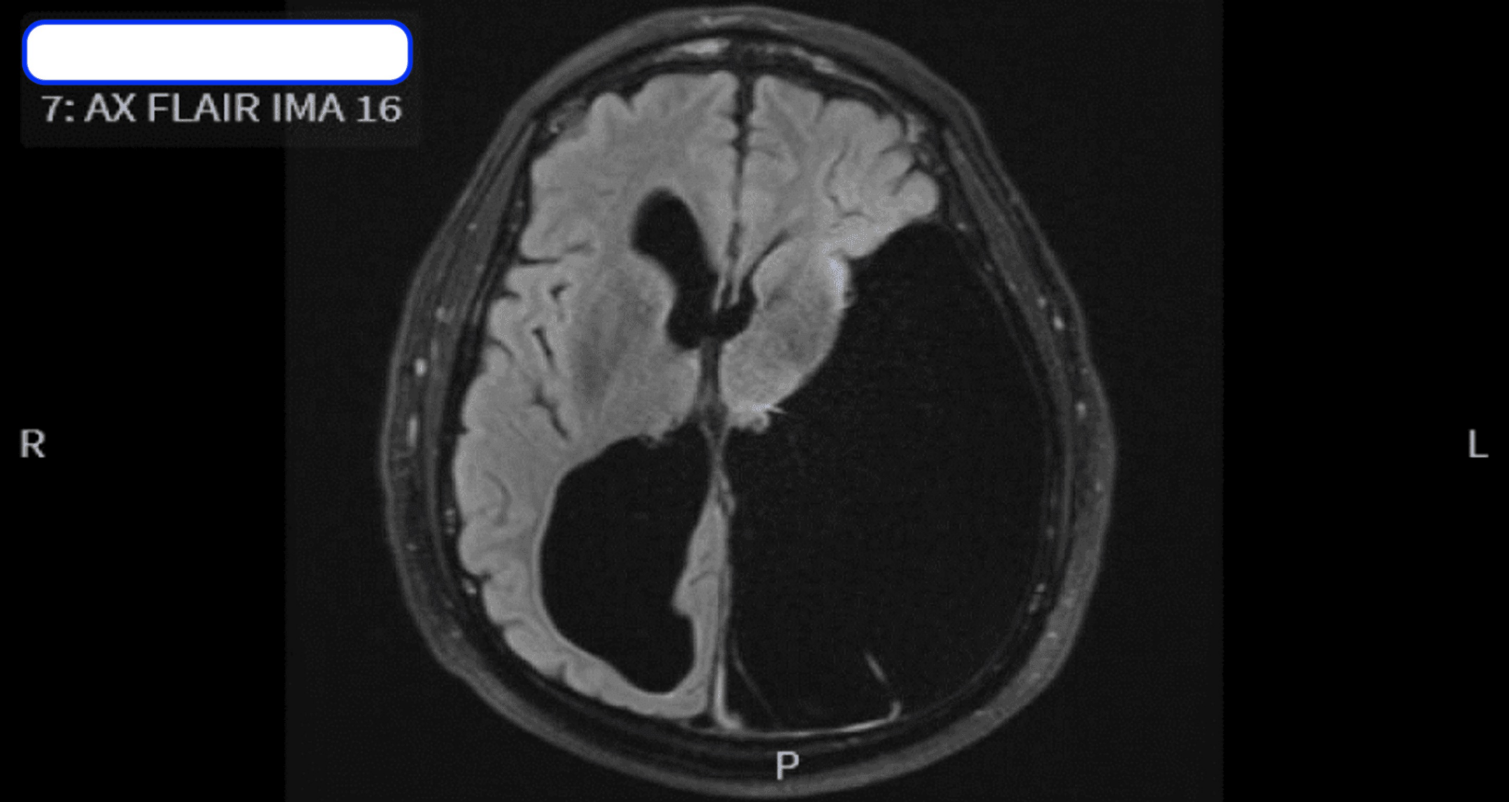
MRI brain imaging displayed a large porencephalic cyst in the left temporal, frontal, and parietal occipital lobes, communicating with the left lateral ventricle, and a chronically dilated right lateral ventricle.

Despite normalization of heart rate and blood glucose, he remained hypothermic requiring ongoing AER. Testing for Human Immunodeficiency Virus (HIV), Rapid Plasma Reagin (RPR), Vitamin B12, folate, urinalysis, urine toxicology, and blood cultures yielded unremarkable results. An EEG revealed focal structural dysfunction over the left hemisphere, with moderate diffuse encephalopathy. MRI brain displayed a large porencephalic cyst in the left temporal, frontal, and parietal occipital lobes, communicating with the left lateral ventricle, and a chronically dilated right lateral ventricle with no acute abnormalities (Figure 1). Neurology consultation determined that the congenital structural brain defect was less likely contributing to the hypothermia. 

The patient's metabolic panel and functional status returned to baseline following two weeks of hospitalization. However, he continued to experience intermittent episodes of hypothermia, with a core body temperature of less than 35°C. Psychiatry was consulted to exclude antipsychotic-induced hypothermia. They recommended transitioning the patient from risperidone to aripiprazole, with gradual titration over the course of one week. Subsequently, the patient showed improvement, and the hypothermic episodes resolved. Upon achieving stability, the patient was discharged back to the nursing home on a consistent dosage of aripiprazole. His mental status remained stable, and no new instances of hypothermic episodes were documented.

## Discussion

Hypothermia is defined as a decline in core body temperature below 35°C and is typically classified, depending on the etiology, into primary and secondary hypothermia. Primary hypothermia is caused by direct exposure to cold environments, while secondary hypothermia is mainly due to internal causes that can lead to impaired thermoregulation, increased heat loss, or decreased heat production ​[7]. Drug-induced secondary hypothermia has been documented in the literature across several drug classes, with APD drawing attention due to the lethality of this adverse effect. 

Although the mechanism behind APD-induced hypothermia is still not fully understood, it is proposed that 5HTa2 receptor blockade may be associated with hypothermia ​[3]. Atypical antipsychotics have a stronger binding affinity for 5HTa2 receptors than for D2 receptors, which contrasts with typical antipsychotics that function mainly by blocking dopamine receptors ​[8]. That said, atypical antipsychotics are in general more associated with APD-induced hypothermia. Furthermore, alpha-adrenergic receptor blockade may also contribute to hypothermia by inhibiting peripheral vasoconstriction and shivering mechanism, which increases surface heat loss and decreases heat production, respectively ​[3]. 

Based on our literature review, the association between antipsychotic drugs (APDs) and hypothermia appears to have been first established in 1952 by Laborit et al. when he employed chlorpromazine, a typical APD, as an adjunct to surgical anesthetics due to its temperature-lowering effects ​[9, 10]. Since then, hypothermia has been reported in various typical and atypical APDs, including risperidone, clozapine, olanzapine, pipamperone, ziprasidone, aripiprazole, and zotepine ​[6]. Until 2019, at least 74 cases of APD-induced hypothermia have been reported ​[11]. However, the United States Food and Drug Administration (FDA) and World Health Organization (WHO) report a much higher incidence with risperidone being responsible for approximately 27% of the cases ​[2,3]. 

Zonnenberg et al. documented 57 cases of hypothermia related to APD use, with 10 cases (14%) linked to risperidone use and 24 cases (42%) not associated with initiation of an APD or increased dosage, which aligns with our case in which risperidone-induced hypothermia occurred without recent dose changes ​[6]. However, this is not the usual scenario, as most of these cases occur a few hours to a few days following a recent administration or change in the dose of an APD ​[2]. Few reports of patients on a stable dose of risperidone, as in our patient, have been published ​[12-16]. However, these reports included recent exposure to a predisposing factor such as a cold environment, infection, renal failure, or drugs. What makes our case unique is that hypothermia developed with no recent introduction of identifiable predisposing factors. 

Presentation varies according to the level of hypothermia, with the mildest forms featuring subtle and non-specific symptoms such as apathy, impaired judgment, and maladaptive behavior ​[5]. Thus, the diagnosis can be overlooked by physicians since most ADP-induced hypothermia patients present with mild stages ​[2]. In addition to that, patients who take antipsychotics might already have a baseline state of drowsiness and apathy. 

Managing hypothermia depends on the clinical severity. The severity of hypothermia is traditionally categorized into mild, moderate, severe, and profound based on the body's core temperature ​[5]. If such measurement is not readily accessible, then the Swiss staging system can be utilized. It integrates vital signs assessment and divides hypothermia into stages I through IV [4]. 

Based on the severity, interventions can range from passive external rewarming such as covering with blankets, to active external rewarming like external heating methods, and in severe cases, might necessitate active internal rewarming, including warmed isotonic crystalloid or application of extracorporeal circulation techniques ​[17]. Discounting the drug, if possible, is recommended during rewarming, especially if the patient did not respond to the primary management as in our case ​[6,18]. However, if additional predisposing factors for hypothermia are present, we may keep the drug on a lower dose with continuous monitoring of the patient ​[6]. 

Upon establishment of the diagnosis of hypothermia in our patient, it was imperative to try and identify the underlying cause of the hypothermia in order to manage it appropriately. Our patient had a Naranjo Adverse Drug Reaction Probability Scale in the probable range (5 - 8), but the causality relationship was not initially suspected due to the rarity of risperidone causing hypothermia in the absence of recent dose adjustment. Therefore, we intended to present our case to illustrate the importance of considering various etiologies of hypothermia and ways of managing it. As our case illustrated, such a complication might arise even when the patient has been taking a stable dose of the same medication for years.

## Conclusions

APD-induced hypothermia is a rare adverse effect that requires a full assessment of suspected predisposing factors, vital signs, and most importantly, core body temperature. Even though moderate to severe cases could present as neurological and hemodynamic instability, mild cases can present as subtle symptoms requiring a higher level of suspicion. Thus, it is recommended to monitor body temperature and vital signs regularly for patients on antipsychotics, even if they are on a stable dose and no known predisposing factors are present.
